# The Usefulness of Cellular Immune Inflammation Markers and Ultrasound Evaluation in the Assessment of Disease Activity in Patients with Spondyloarthritis

**DOI:** 10.3390/jcm12175463

**Published:** 2023-08-23

**Authors:** Bożena Targońska-Stępniak, Krzysztof Grzechnik

**Affiliations:** 1 Department of Rheumatology and Connective Tissue Diseases, Medical University of Lublin, Jaczewskiego 8, 20-059 Lublin, Poland; 2 Department of Rheumatology and Connective Tissue Diseases, Independent Public Teaching Hospital No. 4, Jaczewskiego 8, 20-059 Lublin, Poland; krzysiek.grzechnik@gmail.com

**Keywords:** spondyloarthritis, cellular immune inflammation markers, ultrasound examination

## Abstract

Background: The systemic inflammation response index (SIRI) and systemic immune-inflammation index (SII) have been introduced as inflammatory markers and predictors of poor prognosis in cancer and cardiovascular diseases. An appropriate evaluation of disease activity in spondyloarthritis (SpA) might be challenging. The purpose of this study was to evaluate the usefulness of cellular immune inflammation markers and ultrasound (US) evaluation of entheses and joints in the assessment of disease activity in SpA patients. Methods: This cross-sectional study involved patients with SpA (62 axial SpA, 38 peripheral SpA, pSpA). The clinical data of both tender, swollen joint counts, erythrocyte sedimentation rate, C-reactive protein, white blood cell counts, and disease activity using Bath Ankylosing Spondylitis Disease Activity Index (BASDAI) and Disease Activity Index for Psoriatic Arthritis (DAPSA), were recorded. The SIRI, SII, neutrophil-to-lymphocyte ratio (NLR), platelet-to-lymphocyte ratio (PLR), and lymphocyte-to-monocyte ratio (LMR) were calculated. US examination was performed (22 small joints, Achilles tendon, and plantar aponeurosis for enthesitis). Results: The SII, SIRI, NLR, and PLR were higher, and LMR was lower in patients with high disease activity (BASDAI > 4). Higher SII was observed in pSpA patients with moderate/high disease activity (DAPSA > 14). The SIRI was correlated with clinical and laboratory parameters of disease activity. The SII was correlated with US parameters in pSpA. Higher SII and NLR values were found in patients with signs of activity compared with no activity in the US of peripheral joints. There were no associations with US changes in entheses. Conclusions: The results of this study point to the value of SIRI and SII as biomarkers of disease activity in patients with SpA. The SII was associated with synovitis in the US of the peripheral joints.

## 1. Introduction

Spondyloarthritis (SpA) is a set of distinct inflammatory diseases that share a common genetic background and several clinical manifestations. These diseases include ankylosing spondylitis (AS), psoriatic arthritis (PsA), reactive arthritis (ReA), inflammatory bowel diseases (IBD)-related arthritis, and undifferentiated SpA (USpA). They primarily affect sacroiliac joints and the spine. High disease activity is associated with pain, stiffness, and bone formation, resulting in progressive ankylosis of the axial skeleton. Peripheral joint involvement and enthesitis are common manifestations of SpA. Extra-articular symptoms may also occur (e.g., psoriasis, uveitis, IBD, cardiovascular complications, osteoporosis) and are associated with increased mortality [1,2,3].

The two phenotypes of SpA have been distinguished, axial (axSpA) and peripheral (pSpA), depending on the predominant symptomatology. The prevalent sacroiliitis and the spine involvement define the axSpA. The pSpA is diagnosed when arthritis, especially in large joints of lower extremities, enthesitis, or dactylitis, predominates the clinical presentation [4,5].

In clinical practice, erythrocyte sedimentation rate (ESR) and C-reactive protein (CRP) are usually obtained to evaluate the disease activity. However, the specificity and sensitivity of these biomarkers are low. In addition, elevated values of ESR and/or CRP are observed in about 40–50% of patients with AS [6].

The hematological markers of systemic inflammation, neutrophil-to-lymphocyte ratio (NLR), platelet-to-lymphocyte ratio (PLR), lymphocyte-to-monocyte ratio (LMR), or monocyte-to-lymphocyte ratio (MLR) were estimated to be highly sensitive markers of inflammation in diseases such as cancer, neurological or cardiovascular conditions, systemic autoimmune rheumatic diseases [7,8,9]. They were also examined as indicators of systemic inflammation in patients with SpA [10,11,12,13,14,15], however, with conflicting results. The NLR value was higher in patients with axSpA than in controls and exhibited a correlation with CRP [10,13,15], or there was no significant difference between AS patients and controls [9,11]. The PLR value was significantly higher [9,12,13,15], and there was no significant difference between patients with AS and controls [11]. The NLR was reported to be a marker for predicting the therapeutic effect and persistence of anti-tumor necrosis factor-α (TNF-α) treatment [14]. The MLR was elevated in AS patients compared with non-radiographic axSpA (nr-axSpA) and was positively correlated with inflammatory parameters (CRP, ESR) and spinal movements [15]. The LMR value was higher in patients with axSpA than in controls and was reported as a marker for the evaluation of disease activity and sacroiliitis stage in X-ray [13].

Recently, novel cellular immune inflammation markers based on circulating immune-inflammatory cells have been developed as indicators of systemic inflammatory response [7,16]. The systemic immune-inflammation index (SII) (NLR × platelets) integrates values of NLR and PLR into one single parameter, considering three blood cell populations (neutrophils, lymphocytes, and platelets). Initially, SII was used in patients with hepatocellular carcinoma [17] and later, in other cancer settings, as an inflammation-based prognostic marker [18,19]. However, SII is also being investigated in cardiovascular (CV) [20,21] or autoimmune diseases, such as rheumatoid arthritis (RA), antineutrophil cytoplasmatic antibody-associated vasculitis (AAV), Behcet’s disease [16,22,23], psoriasis, and PsA [24,25], SpA [26,27]. The SII is reported as a prognostic marker in patients with psoriasis and PsA, as well as a new tool indicating activity in patients with RA [16,22,24,25].

The systemic inflammation response index (SIRI) (NLR × monocytes) is based on three other blood cell populations (neutrophils, lymphocytes, and monocytes). Initially, SIRI was used as a prognostic indicator in cancer, CV diseases [21,28,29,30], coronavirus disease (COVID-19), and RA [16]. According to the best knowledge, SIRI was not assessed in patients with SpA.

Imaging methods, including ultrasound (US) and magnetic resonance (MR), have an important role in the management of SpA patients. These methods may be used to monitor inflammatory activity, particularly synovitis, and enthesitis in pSpA. The conflicting data have been reported in the literature with regard to associations between grey-scale (GSUS) or power Doppler (PDUS) changes in the lower limb entheses and inflammatory parameters, indicating significantly positive correlations or no correlation [31].

The aim of this study was to estimate the usefulness of cellular immune inflammation markers and ultrasound (US) evaluation of entheses and joints in the assessment of disease activity in patients with SpA.

## 2. Materials and Methods

### 2.1. Study Population

This cross-sectional study included 100 consecutive patients with SpA (AS and PsA), treated in the Department of Rheumatology and Connective Tissue Diseases, Medical University of Lublin, Poland. Patients with AS fulfilled the 1984 modified New York classification criteria [32], and those with PsA fulfilled the 2006 classification criteria of psoriatic arthritis (CASPAR) [33]. This study’s patients were classified as axSpA (predominant sacroiliitis, spine involvement) or pSpA (predominant peripheral arthritis, enthesitis, or dactylitis).

This study was performed according to the Declaration of Helsinki and was approved by the Ethical Committee of the Medical University of Lublin (approval number KE-0254/21/2020). All patients gave written informed consent prior to being included in this study.

### 2.2. Clinical and Laboratory Assessment

The clinical data were collected through precise interviews, medical history, self-reported questionnaires, and physical examinations.

In patients with axSpA, disease activity was evaluated by using Bath Ankylosing Spondylitis Disease Activity Index (BASDAI) [34], which includes six 10 cm horizontal visual analog scales (VAS) in order to assess the intensity of fatigue, spinal pain, peripheral joint pain, tenderness of entheses, and morning stiffness (range 0–10). The cut point for high disease activity was BASDAI > 4. An assessment of functional ability was performed according to the Bath Ankylosing Spondylitis Functional Index (BASFI) [35], which consisted of 10 questions regarding physical activity and was answered on a 10 cm horizontal VAS. The mean of all answers is the result of the BASFI score (range 0–10) [35].

In patients with pSpA, disease activity was evaluated by using the Disease Activity Index for Psoriatic Arthritis (DAPSA), calculated with a 68/66 joint counts scale (tender joint count (TJC), swollen joint count (SJC), patient global assessment (PGA) by VAS, patient pain assessment by VAS, and CRP concentration (mg/dL) [36]. The cut point for remission is the value ≤ 4, and for low disease activity, the values are between 5 and 14.

The ability to perform daily activities was assessed according to a modified Health Assessment Questionnaire (M-HAQ), with a range of 0–3 (a score of 0 presenting no impairment of function) [37].

Blood samples were taken after overnight fasting. Blood tests performed in all patients included ESR, CRP, and complete blood cell count (CBC). The samples were analyzed with ADVIA 2120i System automated cell counter (Siemens, Munich, Germany). The NLR was calculated as a ratio of peripheral blood neutrophil count/lymphocyte count; PLR peripheral blood platelet count/lymphocyte count; LMR peripheral blood lymphocyte count/monocyte count; SII: (neutrophil × platelet count)/lymphocyte count; SIRI: (neutrophil × monocyte count)/lymphocyte count.

### 2.3. Ultrasound Imaging of Joints and Entheses

The US imaging of joints was made by applying a machine (MyLab25 Gold, Esaote, Genova, Italy) with an 18 MHz broadband high-frequency linear array transducer.

In patients with pSpA, peripheral joints assessment was conducted, including 22 bilateral joints: 2 wrists; 10 metacarpophalangeal (MCP); 8 hand proximal interphalangeal (PIP); and 2 thumb interphalangeal (IP). Images were taken according to the EULAR recommendations with a longitudinal scan, obtained using either a dorsal view (wrists) or a dorsal or volar view (MCPs, PIPs, thumb Ips) [38]. For each joint, an evaluation of GSUS and PDUS was carried out. For the PDUS option, the pulse repetition frequency was configured to 700 kHz, and the gain was increased to the maximum level, not generating random noise. The following scoring system was applied to assess synovitis:

1. Semi-quantitative grey scale (GS) for grading synovial hypertrophy (0–3) in each joint:
  - Grade 0: normal joint with no synovial hypertrophy;
  - Grade 1: synovial hypertrophy up to the level of the horizontal line connecting the bone surfaces of an examined joint;
  - Grade 2: synovial hypertrophy extending beyond the joint line but with the upper surface flat to the underlying bones;
  - Grade 3: synovial hypertrophy extending beyond the joint line but with the upper surface convex to the underlying bones;
2. Power Doppler ultrasound (PDUS) semi-quantitative scale (0–3) in each joint:
  - Grade 0: no Doppler activity;
  - Grade 1: up to three single Doppler spots, or up to one confluent spot and two single spots, or up to two confluent spots;
  - Grade 2: greater than grade 1 but <50% Doppler signals in the total GS background;
  - Grade 3: greater than grade 2 and >50% Doppler signals of the GS background [38].

The highest score from the dorsal or volar view of MCPs, PIPs, and thumb IPs was taken for statistical analysis. Then, we calculated GSUS and PDUS scores by summing scores obtained as a result of an assessment of individual joints (range 0–66). The global score was calculated by summing the GSUS and PDUS scores of all examined joints (range 0–132) [39].

In patients with axSpA, an assessment of the Achilles tendon and plantar aponeurosis for enthesitis (active or chronic) was performed. According to OMERACT US definitions, increased thickness, hypoechogenicity, and Doppler activity of the enthesis were considered to be signs of active enthesitis, while bone erosions at the insertion of the enthesis, enthesophytes, and intratendinous calcifications were considered as symptoms of chronic enthesitis [40].

### 2.4. Statistical Analysis

Categorical data were presented as absolute numbers and percentages. The Kolmogorov–Smirnov test was used to assess the normality of data distribution. Continuous variables with a normal distribution were presented as the mean ± standard deviation (SD). Continuous variables which did not follow normal distribution were presented as the median with interquartile range (IQR). The Student’s *t*-test or nonparametric Mann–Whitney U test were used in order to compare the continuous variables in subgroups of patients. Correlation between the quantitative variables was assessed by Spearman’s or Pearson’s correlation test. The multiple linear regression analysis was conducted with those variables which showed statistically significant association with specific parameters. The *p*-value < 0.05 was considered significant. The Bonferroni correction was used when several correlations were performed simultaneously. All data analyses were performed using the StatSoft STATISTICA 13.3 application.

## 3. Results

### 3.1. Characteristics of This Study’s Group

This study consisted of 51 patients with AS and 49 patients with PsA. The axSpA was found in 62 patients and pSpA in 38 patients (Table 1). Male patients constituted about 2/3 of this study’s group. Nearly ¾ of all the patients were human leukocyte antigen B27 (HLA-B27)-positive (almost all patients with AS), and 93% were IgM rheumatoid factor (RF-IgM)-negative. The disease duration ≥ 10 years was noted in about 40% of cases. Extra-articular manifestations (anterior uveitis, inflammatory bowel disease, heart conduction disturbances, valvular heart disease, osteoporosis) were observed in almost 1/3 of the patients (Table 1).

When evaluating this study’s group, NSAIDs were used in 93 patients, synthetic diseases modifying anti-rheumatic drugs (DMARDs) (sulfasalazine, methotrexate, leflunomide, cyclosporine) in 61 patients, and biological DMARDs (anti-TNFα) in 46 patients. Low-dose glucocorticoids (GC) (prednisone ≤ 10 mg/day) was used in 16 patients (Table 1).

High disease activity (BASDAI ≥ 4) was noted in about 60% of patients with axSpA (Table 2).

In patients with pSpA, remission/low disease activity (DAPSA ≤ 14) was observed in 34% of cases. The Global score = 0 (in the US imaging of peripheral joints) was found in 13% of cases (Table 2).

The value of DAPSA was correlated with US imaging parameters of peripheral joints, GSUS score (R = 0.36, *p* = 0.05), PDUS (R = 0.55, *p* = 0.002), and Global score (R = 0.41, *p* = 0.03).

No correlation was noted between BASDAI and US parameters of axSpA.

### 3.2. Differences between Patients with axSpA and pSpA

The significant differences between axSpA and pSpA patients included age (respectively, 40.0 ± 9.6 vs. 46.1 ± 13.3); M-HAQ value (0.84 ± 0.58 vs. 1.5 ± 0.86), and hemoglobin concentration (14.2 ± 1.5 vs. 13.2 ± 1.6).

There were no significant differences between the two groups with regard to inflammatory parameters, SII, SIRI, NLR, PLR, and LMR.

### 3.3. The SII and SIRI Values in Defined Groups of Patients with SpA

The SII value was significantly higher in patients with BASDAI > 4 when compared with those of BASDAI ≤ 4, in patients with DAPSA > 14 vs. DAPSA ≤ 14, in patients with disease duration ≥ 10 years vs. <10 years, and in patients with the current GC treatment vs. no GC (Table 3).

The SIRI value was significantly higher in patients with BASDAI > 4 when compared with those with BASDAI ≤ 4 and in those with the current GC treatment vs. no GC (Table 3).

**Figure 1:**
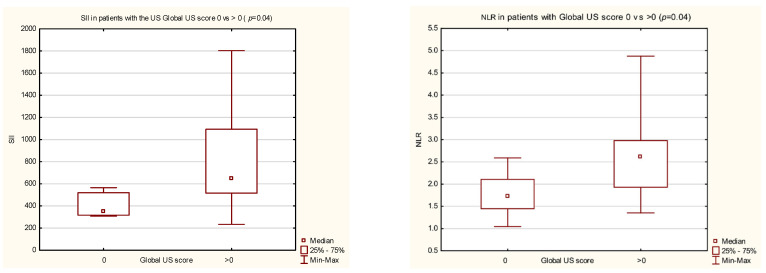
SII and NLR values in patients with Global US score 0 vs. >0.

The SII value was significantly higher in patients with the Global US score > 0 vs. score = 0 (649.3 (516.7–1093.4) vs. 349.6 (316.5–520.5), *p* = 0.04) (Figure 1).

No other differences were observed with regard to disease activity, laboratory, and US parameters or methods of treatment.

### 3.4. The NLR, PLR, and LMR Values in Defined Groups of Patients with SpA

The NLR value was significantly higher in patients with a Global US score > 0 vs. score = 0 (2.6 (1.9–5.0) vs. 1.7 (1.4–2.1), *p* = 0.04) (Figure 1).

Significantly higher values of NLR, PLR, and lower LMR were observed in patients with BASDAI > 4 when compared with those with BASDAI ≤ 4 (Table 4).

The NLR value was significantly higher in patients with the current GC treatment vs. no GC, and the LMR value was significantly higher in patients performing regular physical activity vs. no regular activity (Table 4).

No other differences were observed with regard to disease activity, laboratory and US parameters, or methods of treatment.

### 3.5. Relationships between SII, SIRI, NLR, PLR, LMR, Disease Activity Markers, and US Parameters

Positive correlations were found between SII, SIRI, NLR, and PLR, as well as negative for LMR with BASDAI, BASFI, VAS back pain, CRP, and ESR (Table 5).

The positive associations were noted between SII and DAPSA and US parameters (GSUS, Global score) (Table 5).

No associations were found with parameters of US imaging of the entheses.

In the multiple linear regression analysis, significantly positive associations were found for SIRI with BASFI, M-HAQ, and CRP (Table 6). Positive correlations were confirmed between SII and US parameters (GSUS, Global score) (Table 6).

The positive correlations were confirmed between PLR and inflammatory parameters (CRP, ESR) (Table 6).

## 4. Discussion

According to the best knowledge, this study is the first to report positive correlations between SIRI and disease activity parameters (both clinical and laboratory) in patients with SpA. This study is also the first to report positive associations between SII and US parameters of disease activity (GSUS, Global score) of peripheral joints in patients with pSpA.

In this study, it was shown that all the cellular inflammation markers were correlated with clinical and laboratory parameters of disease activity. The SII, SIRI, NLR, and PLR were significantly higher, and LMR significantly lower, in axSpA patients with high disease activity (BASDAI > 4). Higher SII values were observed in pSpA patients with moderate/high disease activity (DAPSA > 14).

Moreover, SII was correlated with US parameters in patients with pSpA. The median values of SII and NLR were significantly higher in patients with signs of any activity when compared with those with no activity in the US imaging of peripheral joints. However, no significant association was noted with US changes in the entheses in SpA patients.

Significantly higher values of SII, SIRI, and NLR were found in patients with the current GC treatment when compared with patients with no GC treatment. The SII was higher in patients with a disease duration ≥ 10 years. These results might be attributed to the long-standing, active disease, which requires GC treatment.

The LMR value was significantly higher in patients performing regular physical activity when compared with no regular activity. According to the literature, in patients with multiple sclerosis, high-intensity-interval training reduced both NLR and SII [41]. However, there are no reports with regard to patients with SpA. In the study of a healthy population, a negative association between LMR and physical activity was observed [42].

The total WBC count is a useful measure to assess the state of inflammation, but it fails to demonstrate different WBC subsets. The cellular immune inflammation markers provide diverse insight into the inflammatory process considering different immune cell populations. Moreover, these indices are easily accessible, simple, cheap tools that might be helpful in the assessment of inflammatory process activity.

The NLR was first introduced as a prognostic marker in cancer patients and later was used in inflammatory and autoimmune diseases as a prognostic marker indicating long and short-term risks of heart failure, arrhythmia, and mortality in different populations of patients [9,14,15,41]. Platelets not only play a central role in thrombosis, but they exhibit different pro-inflammatory properties; therefore, PLR may be used alternatively to NLR as an inflammation marker [14]. Lymphocytes participate in immune identification and response. In systemic inflammation, the increase in neutrophil count usually corresponds with the lymphocyte count decrease [26]. Monocytes have a significant role in the activation of the innate immune system; therefore, LMR might also be an important inflammatory marker [11].

The novel cellular immune inflammation markers, SII and SIRI, integrate three blood cell populations into a single parameter. These two composite indices seem to be more stable than individual blood count results and not susceptible to various factors, such as dehydratation or over-hydration [20]. The SII and SIRI were first introduced as independent predictors of unfavorable prognosis in cancer, which can be represented by the systemic inflammatory response. Further studies showed that higher neutrophils, monocytes, and lower lymphocyte counts were associated with a higher risk of CV diseases [14,43]. According to a recent report, the adult population with SII higher than 655.56 had higher all-cause and CV mortality (hazard ratio, HR ~1.3) than those with SII < 335.36. Adults with SIRI higher than 1.43 had a higher risk of all-cause and CV death (HR ~1.4) than those with SIRI < 0.68. The risk of all-cause death increased apparently in adults over 60 years of age [21]. In older adults with hip fractures and surgeries, poor all-cause mortality was reported in relation to higher SII. It was estimated that each increase of 100 units of SII was associated with an 8% increased risk of death at 1-year follow-up [44].

The mean values (95% reference intervals) of the indices were determined according to the population-based study, NLR 1.76 (0.83–3.92), PLR 120 (61–239), and SII 459 (189–1168). Values of NLR and SII increased with age, which might be attributed to the higher prevalence of pathologies associated with inflammation in older age. The PLR decreased with age, which corresponded with a decrease in platelets in the older population. Higher PLR and SII values were reported in females, and NLR was higher in males [45]. In this study, a higher SII value was noted in patients with long-standing disease; however, no age or sex differences were noted.

The circulating blood cells play a crucial role in SpA pathogenesis and the disease course. Autoimmune rheumatic diseases and SpA are characterized by aberrant activation of innate and adaptive system immune cells [2]. The presence of neutrophils was demonstrated in healthy spinal peri-enthesal bone. Neutrophils from the bloodstream move to sites of inflammation in SpA, including peripheral joints, entheses, skin, eyes, and bowels, where they produce cytokines (e.g., interleukin 17, IL-17), chemokines, and activate other immune cells [1]. In animal studies, neutrophils were found in the early inflammatory infiltrate at axial and peripheral enthesal sites [3]. In active SpA, neutrophil infiltration was correlated with CRP and ESR as a sign of the relationship between systemic and local inflammation [1]. Lymphocytes, platelets, and monocytes are reported to have a significant role in the inflammatory process in the course of SpA. In the first study exploring the role of SII in AS, the SSI was found to be a relevant indicator of disease activity, with a cut-off value of 513.2 for the diagnosis of active AS [26]. In the first study investigating the role of SII in psoriasis and PsA, the SII was higher in psoriatic patients with arthritis vs. no arthritis [24].

In this study, the median value of SII was > 600 and SIRI > 1.0, suggesting active disease. Both SII and SIRI values were significantly higher in patients with high disease activity and correlated with inflammatory parameters. Our study is the first research reporting the role of SIRI in SpA.

Enthesitis is one of the main features of SpA. The clinical assessment of enthesitis seems to be inadequate due to poor specificity, and the US examination might improve the assessment of enthesitis. However, US diagnostic accuracy is questionable due to the high prevalence of US enthesitis in healthy subjects [46]. In this study, we found no associations between cellular inflammation markers and US changes in entheses.

The SII value was associated with synovitis in US imaging of peripheral joints.

This study has some limitations. First, the relatively low study population. The higher number of cases included in this study could improve the statistical analysis. Second, the cross-sectional character of this study is associated with some limitations in the results. Third, we used BASDAI for the assessment of the disease activity. It would be useful to apply ASDAS and compare the results. Fourth, US examination for enthesitis was performed only in Achilles tendon and plantar aponeurosis; a higher number of entheses might be examined.

Our study has some strengths. First, according to the best knowledge, this is the first study to evaluate relationships between SIRI and disease activity in patients with SpA. Second, this study is also the first to report positive associations between SII and US parameters of synovitis of peripheral joints in patients with pSpA. Third, patients included in this study came from everyday clinical practice and were not selected for this study. Fourth, the US examination was performed by the same experienced physician. Fifth, all laboratory assessments are cheap and available and, therefore, may be used in everyday clinical practice.

## 5. Conclusions

In this study, we found significant relationships between SIRI and clinical and laboratory parameters of disease activity in patients with SpA. The SII correlated with the US synovitis markers. These results point to the value of SIRI and SII as biomarkers of disease activity in patients with SpA. Further research is required to confirm this statement.

## Figures and Tables

**Table 1:** Characteristics of patients with SpA.

Data	Results (n = 100)
Age, years	42.3 (±11.4)
Gender, female/male (n,%)	38 (38.0)/64 (64.0)
Disease duration, years	8.0 (3–16)
Disease duration ≥ 10 years (n,%)	42 (42.0)
AS patients PsA patients	51 (51.0) 49 (49.0)
Patients with axSpA Patients with pSpA	62 (62.0) 38 (38.0)
HLA-B27 (+) SpA patients (n,%) HLA-B27 (+) AS patients (n,%) HLA-B27 (+) PsA patients (n,%)	74 (74.0) 48 (94.1) 26 (53.1)
Positive RF-IgM (n,%)	7 (7.0)
Extra-articular manifestations (n,%)	32 (32.0)
BMI, kg/m^2^	26.6 (24.6–29.8)
Regular physical activity (n,%)	36 (64.0)
Arterial hypertension (n,%)	44 (44.0)
Family history of cardiovascular diseases (n,%)	43 (43.0)
Diabetes (n,%)	2 (2.0)
Current NSAID used (n,%)	93 (93.0)
Current synthetic DMARD used (n,%)	61 (61%)
Current biological DMARD used (n,%)	46 (46.0)
Current low dose GC use (n,%)	16 (16.0)

Values are displayed as mean ± standard deviation (SD), median (IQR), or frequencies with corresponding percentages (%). AS, ankylosing spondylitis; axSpA, axial SpA; BMI, body mass index; DMARD, diseases modifying anti-rheumatic drug; GC, glucocorticosteroid; HLA, human leukocyte antigen; NSAID, non-steroidal anti-inflammatory drug; PsA, psoriatic arthritis; pSpA, peripheral SpA; RF-IgM, IgM rheumatoid factor; SpA, spondyloarthritis.

**Table 2:** Clinical and laboratory parameters in SpA patients.

Data	Results
Laboratory results (n = 100)	
CRP, mg/L	10.7 (1.6–22.4)
ESR, mm/h	20.5 (7–38)
Hemoglobin, g/dL	13.8 (±1.6)
WBC, 10^9^/L	7.0 (±2.6)
PLT, 10^9^/L	282.7 (±84.9)
Neutrophils, 10^9^/L	4.8 (±2.0)
Lymphocytes, 10^9^/L	1.8 (±0.5)
Monocytes, 10^9^/L	0.4 (±0.2)
NLR	2.7 (1.9–3.8)
PLR	153.1 (119.1–205.5)
LMR	4.2 (3.2–5.3)
SII	648.2 (469.0–1148.1)
SIRI	1.1 (0.7–1.7)
M-HAQ	1.1 (±0.8)
Clinical parameters of axSpA activity (n = 62)	
VAS back pain (mm)	48.6 (±26.8)
BASDAI	4.6 (±2.4)
BASFI	4.1 (±2.3)
High disease activity (BASDAI > 4) (n,%)	37 (59.7)
US parameters of axSpA	
Active enthesitis (n,%)	20 (32.3)
Chronic enthesitis (n,%)	15 (24.2)
No signs of enthesitis (n,%)	27 (43.5)
Clinical parameters of pSpA activity (n = 38)	
TJC (68 examined)	9.8 (±8.3)
SJC (66 examined)	4.6 (±3.9)
PGA (VAS), mm	47.8 (±23.4)
Patient pain (VAS), mm	36.8 (±17.3)
DAPSA	24.9 (±14.6)
Remission/Low Disease Activity (DAPSA ≤ 14) (n,%)	13 (34.2)
Morning stiffness, minutes	75.2 (±70.3)
US parameters of pSpA	
GSUS score (hypertrophy)	3 (2–10)
PDUS score	0 (0–2)
Global score	5 (2–12)
Global score = 0 (n,%)	5 (13.2)

Values are displayed as mean ± standard deviation (SD), median (IQR), or frequencies with corresponding percentages (%). BASDAI, Bath Ankylosing Spondylitis Disease Activity Index; BASFI, Bath Ankylosing Spondylitis Functional Index; CRP, C-reactive protein; DAPSA, Disease Activity index for Psoriatic Arthritis; ESR, erythrocyte sedimentation rate; GSUS, Grey Scale Ultrasound; LMR, lymphocyte–monocyte ratio; M-HAQ–modified health assessment questionnaire; NLR, neutrophil–lymphocyte ratio; PLT, platelet count; PDUS, Power Doppler ultrasound; PGA, patient global assessment; PLR, platelet–lymphocyte ratio; SII, systemic immune-inflammation index; SIRI, systemic inflammation response index; SJC, swollen joint count; TJC, tender joint count; VAS, Visual Analogue Scale, WBC, white blood cell count.

**Table 3:** The SII and SIRI values in different groups of SpA patients.

Parameters	SII	*p*-Value	SIRI	*p*-Value
BASDAI ≤ 4 BASDAI > 4	488.6 (304.8–717.8) 938.3 (607.7–1214.1)	<0.001	0.8 (0.5–1.1) 1.4 (0.9–2.3)	<0.001
DAPSA ≤ 14 DAPSA > 14	529.9 (308.3–718.5) 713.8 (537.9–1214.1)	0.02	NS	
Disease duration < 10 years ≥10 years	580.1 (450.1–918.7) 866.6 (537.9–1260.9)	0.04	NS	
No current GC treatment Current GC treatment	597.2 (450.3–1036.5) 1196.9 (904.8–1652.6)	0..02	1.0 (0.6–1.5) 1.9 (0.9–2.9)	0.03

Values are displayed as median (IQR). BASDAI, Bath Ankylosing Spondylitis Disease Activity Index; DAPSA, Disease Activity Index for Psoriatic Arthritis; GC, glucocorticosteroid; SII, systemic immune-inflammation index; SIRI, systemic inflammation response index. NS, non-significant.

**Table 4:** The NLR, PLR, and LMR values in different groups of SpA patients.

Parameters	NLR	*p*-Value	PLR	*p*-Value	LMR	*p*-Value
BASDAI ≤ 4 BASDAI > 4	1.9 (1.3–2.9) 3.2 (2.6–3.9)	<0.001	146.6 (108.4–165.4) 166.2 (136.3–251.3)	0.03	5.3 (3.8–4.9) 3.3 (2.8–4.5)	<0.001
Physical activity regular No regular activity	NS		NS		4.8 (3.4–5.5) 3.9 (3.0–5.0)	0.04
No current GC treatment Current GC treatment	2.6 (1.8–3.4) 3.7 (2.8–5.0)	0.01	NS		NS	

Values are displayed as median (IQR). BASDAI, Bath Ankylosing Spondylitis Disease Activity Index; GC, glucocorticosteroid; LMR, lymphocyte–monocyte ratio; NLR, neutrophil–lymphocyte ratio; PLR, platelet–lymphocyte ratio. NS, non-significant.

**Table 5:** Significant correlations between SII, SIRI, NLR, PLR, LMR, and clinical, laboratory, and US parameters in patients with SpA.

Data/*p*-Value/R	SII	SIRI	NLR	PLR	LMR
BASDAI	<0.001	<0.001	0.002	0.01	<0.001
	0.4	0.39	0.37	0.3	−0.43
BASFI	0.001	<0.001	0.009	NS	<0.001
	0.4	0.45	0.33		−0.47
VAS back pain	<0.001	<0.001	0.002	0.01	<0.001
	0.41	0.4	0.37	0.3	−0.41
DAPSA	0.009	NS	NS	NS	NS
	0.43				
M-HAQ	0.007	0.004	NS	NS	0.008
	0.27	0.28			−0.27
CRP	<0.001	<0.001	<0.001	<0.001	<0.001
	0.6	0.61	0.5	0.35	−0.51
ESR	<0.001	<0.001	<0.001	<0.001	<0.001
	0.43	0.41	0.37	0.38	−0.33
GSUS score	0.01	NS	NS	NS	NS
	0.44				
Global score	0.02	NS	NS	NS	NS
	0.41				

Adjustment for multiple comparisons (Bonferroni correction) has been applied. BASDAI, Bath Ankylosing Spondylitis Disease Activity Index; BASFI, Bath Ankylosing Spondylitis Functional Index; CRP, C-reactive protein; DAPSA, Disease Activity index for Psoriatic Arthritis; ESR, erythrocyte sedimentation rate; GSUS, Grey Scale Ultrasound; LMR, lymphocyte–monocyte ratio; M-HAQ—modified health assessment questionnaire; NLR, neutrophil–lymphocyte ratio; PDUS, Power Doppler ultrasound; PLR, platelet–lymphocyte ratio; SII, systemic immune-inflammation index; SIRI, systemic inflammation response index; VAS, Visual Analogue Scale. NS, non-significant.

**Table 6:** Results of multiple linear regression analyses in patients with SpA.

Data (R2)/ *p*/b Value	SII	SIRI	NLR	PLR	LMR
BASFI (0.21)	NS	0.02	NS	NS	NS
		0.47			
M-HAQ (0.06)	NS	0.04	NS	NS	NS
		0.32			
CRP (0.34)	NS	<0.001	NS	0.008	NS
		0.69		0.4	
ESR (0.32)	NS	NS	NS	0.02	NS
				0.35	
GSUS score (0.19)	0.009	NS	NS	NS	NS
	0.46				
Global score (0.17)	0.01	NS	NS	NS	NS
	0.44				

BASFI, Bath Ankylosing Spondylitis Functional Index; CRP, C-reactive protein; ESR, erythrocyte sedimentation rate; GSUS, Grey Scale Ultrasound; LMR, lymphocyte–monocyte ratio; M-HAQ—modified health assessment questionnaire; NLR, neutrophil–lymphocyte ratio; PLR, platelet–lymphocyte ratio; SII, systemic immune-inflammation index; SIRI, systemic inflammation response index. NS, non-significant.

## Data Availability

All data reported in this study are available upon request by contact with the corresponding author.
